# Hospitals and Asylums of the World

**Published:** 1893-01-28

**Authors:** 


### HOSPITAL ADMINISTRATION.

### "HOSPITALS AND ASYLUMS OF THE WORLD."

Every one must feel that the appearance of the last volumes of this exhaustive book,* with the magnificent portfolio of plans which accompanies them, is a subject for congratulation not only to the author, who, as we learn from the introduction, has expended fourteen years in its preparation, but also to the ever increasing number of those con-

* "Hospitals and Asylums of the World." By HENRY C. BURDETT. Four Vols. Portfolio and Plans complete, £8 8s.; Vols. I. and II. Asylums, £4 10s.; Vols. III. and IV. Hospitals, with Portfolio, £6. (London: The Scientific Press, 140, Strand; and J. and A. Churchill.)

cerned directly or indirectly with hospitals and their management. These volumes deal comprehensively with hospitals as the preceding ones did with asylums, and every branch of this wide subject is treated with a thoroughness of practical attention to detail which have not proved incompatible with the broad and tolerant views of ripe experience. It is this breadth of view which helps to make the work attractive, not merely to the student hunting for information to be found nowhere else, but also to all whose hearts are stirred by the great problem of alleviating human misery. No better corrective to the narrow insularity which regards this problem exclusively from the standpoint of our own age and country can be found than this "History of the Hospitals of the World."

It is ungracious perhaps to sit before a work of this magnitude asking, like Oliver Twist, for more. But the historical portion dealing in a scholarly and succinct style with pre-Christian and mediæval hospitals contains so much valuable and hitherto inaccessible information as to rouse a feeling of regret at its conciseness when the nineteenth century is reached, and the subject branches off to poor law infirmaries and hospitals for infectious diseases. A very valuable portion of this third volume is occupied with the question of hospital revenues. It is a marked feature of the work in dealing with disputed questions, and one which will commend it to persons of all shades of opinion that vague generalities are studiously avoided. Facts and statistics are provided with a precision which should enable everyone to form his own conclusions, while the author's convictions, though not concealed, are never insistently urged.

Very interesting and exhaustive details are supplied with regard to the various systems of nursing. And the review of the hospitals of the world, which absorbs the greater part of this volume, presents a mass of well digested and arranged information truly marvellous in extent. It is this branch of the work of which the introduction states: "The special difficulties of the undertaking have proved sometimes almost insurmountable. The great number of languages which had to be grappled with made it very difficult to deal clearly with every country when information was at length forthcoming. It was not an unusual experience to have a mass of printed matter supplied by a foreign Government, which proved, on examination, to contain very little information of value, and to omit most of the special points about which it was desirable to have accurate facts and figures." Understanding these circumstances, the exact and clear statements presented to the reader will be appreciated at their full value.

The fourth volume is devoted to hospital construction. From the choosing of the site to the admission of the first patient no point is omitted in the building, furnishing, and organisation of every kind of hospital. The lust for building grows from the mere contemplation of these studies of drains and foundations. No more fascinating occupation could be suggested to one of the wealthy "unemployed" than to evolve from these volumes, aided by the beautiful diagrams of the portfolio, the ideal hospital, mounted regardless of expense. It is certain that for the future the difficulties which beset the founding of any such institution have been reduced to a minimum. With such an array of examples accessible, the glaring defects which have too often, even recently, disfigured the costliest public erections will know no excuse.

It remains to notice the full bibliography, betraying in its many pages an amount of patient research only to be fully estimated by those having some experience in the difficulties presented by such a compilation. The index is not the least important point in a work of this size, and we are glad to notice that it is unusually complete and accurate. The printing and general appearance of the volumes, and especially of the exquisitely prepared plans in the portfolio, reflect the greatest credit upon the architects, printers, electrotypers, and publishers. The binders, too, have very skilfully dealt with the plans, some of which must have proved most difficult to handle.

